# Incidentally detected pancake kidney: a case report

**DOI:** 10.1186/s13256-020-02455-0

**Published:** 2020-08-14

**Authors:** Sabyasachi Bakshi

**Affiliations:** 1 Department of General surgery, BSMCH, Bankura, West Bengal PIN-722102 India; 2 Kathghara Lane,Sonatuli, Hooghly, 712103 West Bengal India

**Keywords:** Ectopic kidneys, Pancake kidney, Unascended/low-lying fused kidneys, Empty renal fossa

## Abstract

**Background:**

Congenital anomalies of the urinary system are very common and have extremely varied presentation. Among them, the most rarely found structural anomaly is the pancake kidney. When both kidneys are fused along their medial surfaces to form a round-shaped single renal mass, it is termed as pancake kidney. In this case report, a pancake kidney was incidentally detected in a girl. The majority of individuals who have pancake kidney are usually asymptomatic but surgeons should be aware of coexisting malformation of other organs and its potential risk of developing malignancy.

**Case presentation:**

A 12-year-old Bengali girl presented to our out-patient department with mild, dull aching, lower abdominal pain and dysuria. She had no history of fever, hematuria, menstrual abnormality, pelvic inflammatory disease, or trauma. Urine examination showed traces of albumin and 10–12 pus cells/high-power field. She had normal kidney function test but a digital X-ray of her kidney, ureter, and bladder region failed to demonstrate bilateral renal tissue shadows. Ultrasonography of her whole abdomen showed normal intra-abdominal organs except for empty bilateral renal fossa. A multi-detector computed tomography scan of her whole abdomen revealed one round-shaped mass measuring approximately 9 cm (vertical) × 10 cm (horizontal) in the pelvic cavity. That mass was finally identified as a pancake kidney. She was prescribed antibiotics based on urine culture and sensitivity test that cured her symptoms. She was advised to follow-up regularly in our out-patient department to evaluate her kidney function and to rule out any neoplastic change.

**Conclusions:**

This condition can be managed conservatively, if the individual remains asymptomatic, by regular monitoring of renal function. Surgeons should remain alert for the development of infections, any obstructive manifestations leading to calculus formation, and any malignant changes. The individual should be careful in avoiding trauma to low-lying pelvic kidney. Extensive surgeries should be avoided and only selective procedures should be done so that the patient may lead a normal lifestyle.

## Background

Congenital malformative uropathies rank third among the most common congenital anomalies after cardiac and skeletal defects. Congenital anomalies of the urinary system have extremely varied presentation and may involve the kidney, ureter, bladder, or urethra. There may be different developmental renal anomalies, like renal agenesis and ectopic kidney, and different fusion anomalies (most common being horseshoe-type kidney). The most rarely found structural anomaly (less than 10%) is pancake kidney [1]. When kidneys are fused along with the medial surfaces of each pole forming a ring-like or donut-shaped structure, it is called donut kidney. When both kidneys are extensively fused along the whole medial surface, a disc-shaped or shield-shaped single renal mass is formed, which lacks any intervening septum, it is termed as lump or pancake kidney. Each half of the kidney is drained by its own collecting system which lacks connection with the opposite side. The pelvis of this renal mass is placed anterior, and two ureters usually (in rare cases only single ureter) remain uncrossed and enter the urinary bladder following a normal but shorter path. In this case report, a pancake kidney was incidentally detected in an otherwise healthy girl who was initially found to have bilaterally absent kidneys following ultrasonographic study during treatment of her urinary tract infection. The majority of individuals with pancake kidney are asymptomatic but there may be associated malformation of other systems or organs. However, in the present case, no other abnormalities could be detected and our patient was managed conservatively. The aim of this case report is to get acquainted with the potential traumatic, iatrogenic, and possible neoplastic complications of apparently asymptomatic pancake kidney disease and the literature was reviewed to explore possible etiologies and therapeutic strategies.

## Case presentation

A 12-year-old Bengali girl with no significant previous medical or surgical history, presented to our out-patient department with chief complaint of mild, dull aching, lower abdominal pain and dysuria for last 7 days. She denied any history of fever, hematuria, menstrual abnormality, pelvic inflammatory disease, or trauma. A physical examination revealed a normotensive girl with body mass index (BMI) of 19.5, without any significant finding. Her respiratory system and abdominal examination were unremarkable. Her abdomen was scaphoid with umbilicus in the normal position. No intra-abdominal mass was noticed. There was normal bowel sound with no abdominal or renal angle tenderness. There was neither shifting dullness nor any guarding of abdominal muscles.

**Fig. 1:**
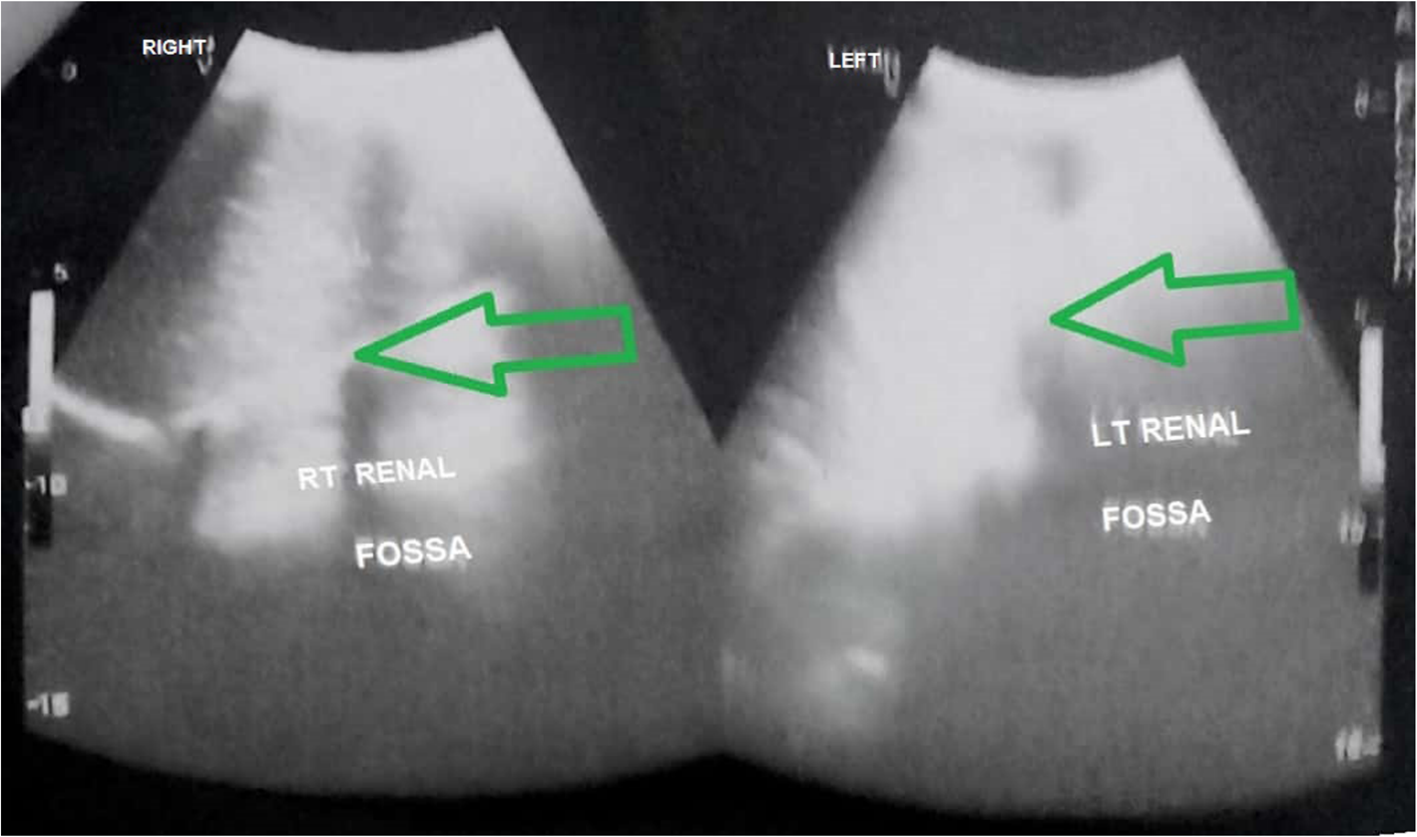
Grey scale ultrasonography scan of the abdomen shows bilateral empty renal fossa (*green arrows*)

**Fig. 2:**
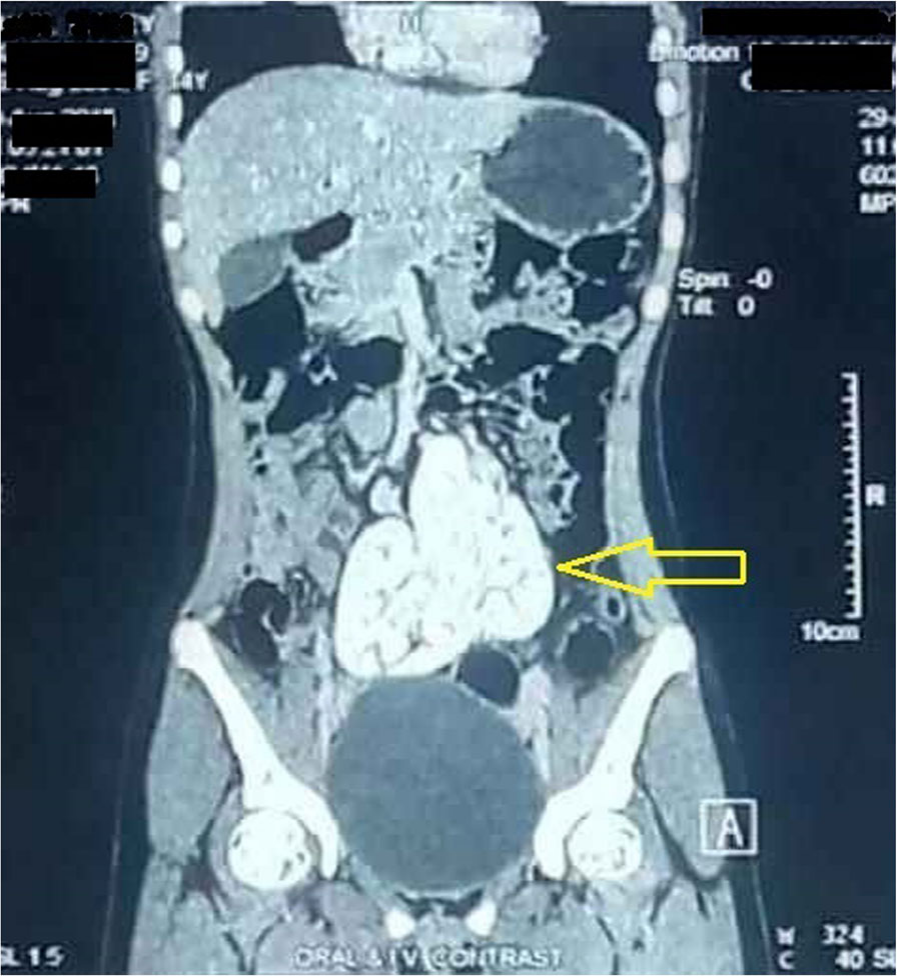
Contrast-enhanced computed tomography (multi-detector computed tomography) scan of abdomen shows pancake kidney, which is placed below the aortic bifurcation in the pelvis with short ureters (*yellow arrow* in coronal section)

**Fig. 3:**
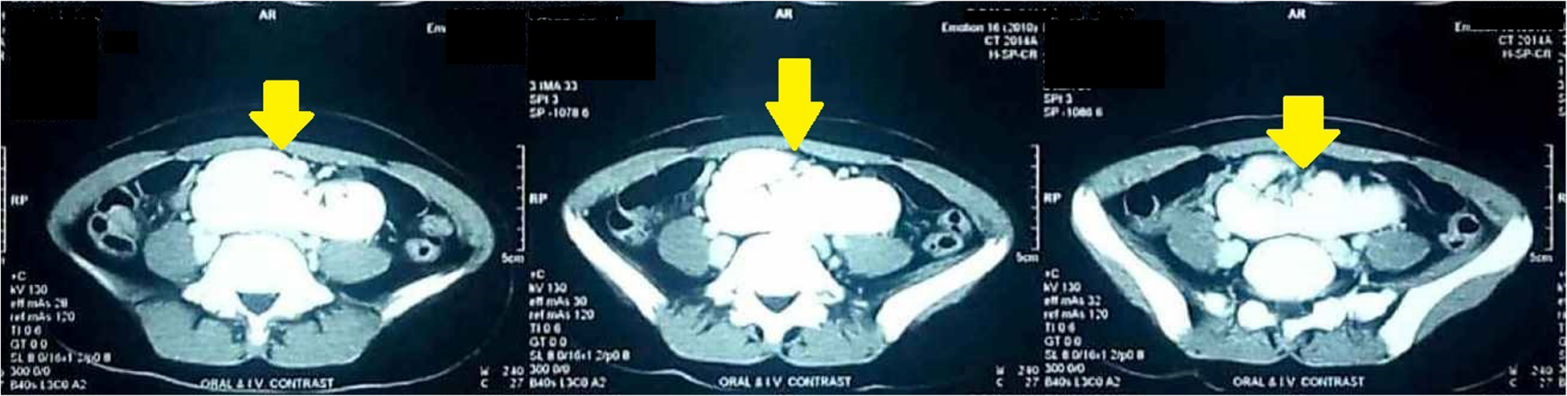
Multi-detector computed tomography scan showing the centrally placed pancake kidney (*yellow arrows* in transverse section)

Microscopic examination of urine showed traces of albumin and 10–12 pus cells/high-power field (HPF) without any red blood cells. She had normal kidney function test (urea, 15.52 mg/dl; creatinine, 0.85 mg/dl) but confusion started when a digital X-ray of her kidney, ureter, and bladder (KUB) region failed to demonstrate bilateral renal tissue shadows. It showed no KUB region calculus also. An ultrasonographic scan of her whole abdomen also added to the pre-existing confusion by revealing no abnormality of intra-abdominal organs except empty bilateral renal fossa (Fig. 1). Ultrasonography (USG) also failed to trace any of the unascended kidneys in bilateral para-vertebral regions in that otherwise healthy girl. Finally, an abdominopelvic multi-detector computed tomography (MDCT) scan (Figs. 2 and 3) showed one round-shaped mass measuring approximately 9 cm (vertical) × 10 cm (horizontal), situated in front of the sacral promontory in the pelvic cavity. That mass was finally identified as two ectopic kidneys malrotated posterolaterally and fused together in the medial aspect in midline anterior to the third, fourth, and fifth lumbar vertebra (L3–L5, below the bifurcation of abdominal aorta) giving rise to a pancake kidney. The corticomedullary differentiation was maintained. The parenchymal enhancement pattern was normal with excretion of contrast material, which was seen bilaterally. There was no evidence of calculus, obstruction, or hydronephrosis. Short, uncrossed, non-dilated ureters were seen, anterior to renal mass, draining separately into the urinary bladder. MDCT clearly showed both the vascular supply and urinary tract anatomy.

She was prescribed antibiotics based on urine culture and sensitivity test that cured her symptoms (fever and dysuria). Although no follow-up guideline is available in the literature, she was advised to follow-up regularly in urology out-patient department to evaluate her kidney function (to be done yearly if asymptomatic) and to rule out any neoplastic change (by routine ultrasonographic scan yearly if asymptomatic and by MDCT if any symptom arises).

## Discussion and conclusions

In 1938, Wilmer first described and classified renal fusion anomalies; the classification was refined and expanded by McDonald and McClellan in 1957 [2]. According to them, renal ectopia is of two types: crossed type and simple/uncrossed type. Crossed renal ectopia may be of four types:

i. Bilateral crossed ectopia of unfused kidneys.
ii. Unilateral crossed ectopia of unfused kidneys.
iii. Bilateral crossed ectopia of fused kidneys – commonest (90%).
iv. Crossed unfused renal ectopia.

Other rare fusion anomalies are sigmoid kidney, L-shaped kidney but, rarely, is there an incidence of familial crossed ectopia. Out of the population, 3.3–11.1% have congenital anomalies of the urinary system, which accounts for nearly 50% of all congenital abnormalities. The overall occurrence of ectopic kidney is 1 out of 400 autopsy cases and among them 85% had fused kidney [3]. In a study, the incidence of prenatal USG-detected crossed renal ectopia was 0.003% in India. The commonest (incidence of 1 in every 700 autopsies and 0.25% in the general population) renal ectopia is horseshoe kidney [4].

Pancake kidney is the rarest type of ectopic fused kidney disease with unknown occurrence. However, Miclaus *et al.* had calculated that 1 out of 65,000–375,000 population may get affected [5]. Pancake kidney has male preponderance (male:female ratio is 2.5:1). The condition may be detected at any age, although the most common age for detection is 30–60 years [6]. Crossed ectopic kidney (second most common fusion abnormality) has varied presentation. In this situation, both the kidneys may be situated on the ipsilateral side in a fused manner (85%), in unfused manner (< 10%), or in extreme rarity it may be bilateral or solitary. The left kidney more commonly (three times commoner than the right kidney) migrates to the opposite side [7]. Commonly, the upper pole of the crossed ectopic kidney fuses with the lower pole of the uncrossed kidney.

**Fig. 4:**
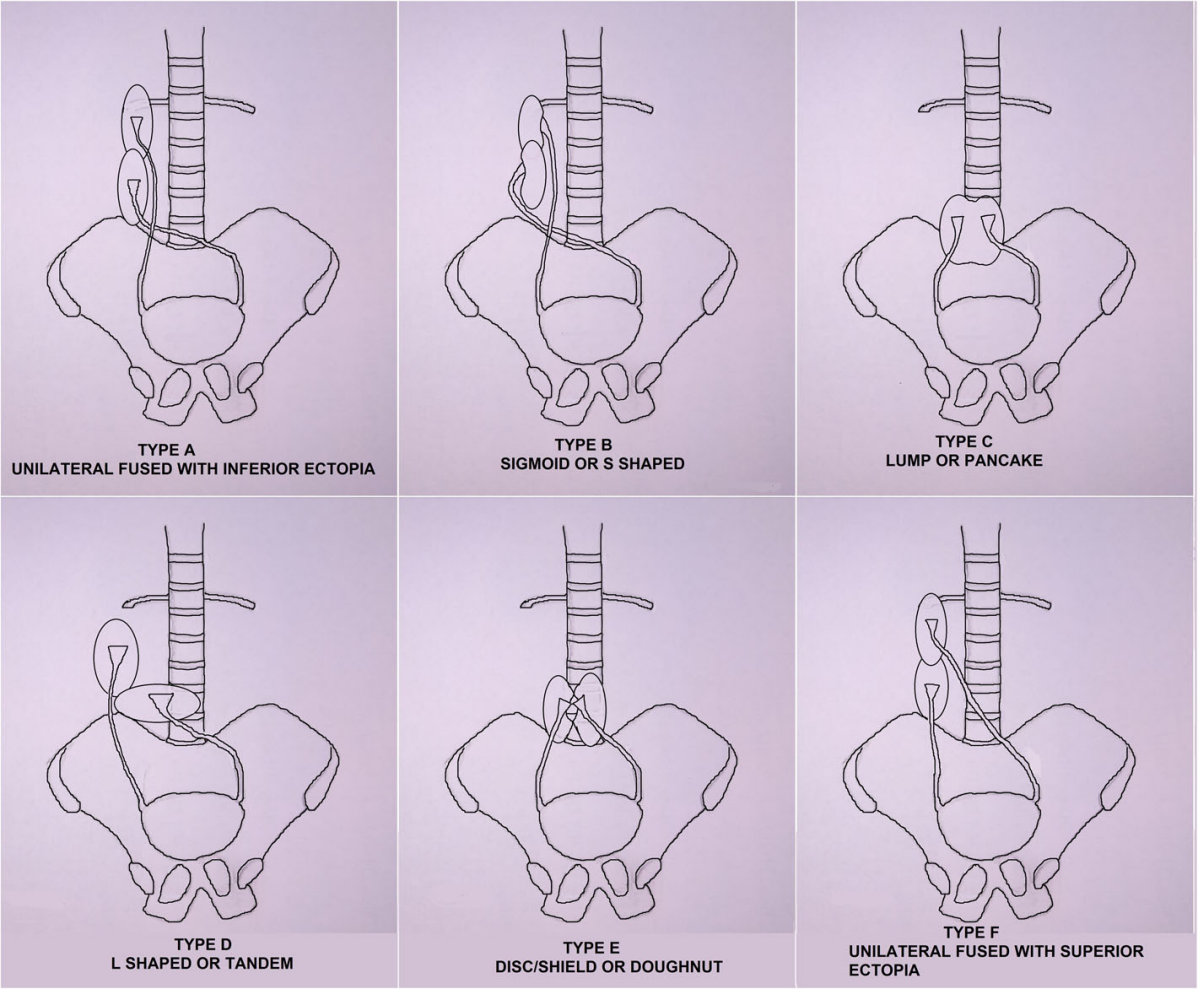
Hand-drawn illustration showing McDonald and McClellan’s classification of crossed fused renal ectopia

McDonald, McClellan *et al.* had classified crossed fused renal ectopia (in decreasing order of frequency) into six categories [8], see (Fig. 4):

i. Type A – Inferior crossed fusion. Here the superior part of the ectopic crossed kidney fuses with the inferior part of the ipsilateral uncrossed kidney. Pelvis of both the kidneys may be located anteriorly.
ii. Type B – S-shaped or sigmoid kidney. Here the ectopic crossed kidney is placed inferiorly with pelvis directed laterally and the ipsilateral uncrossed kidney is placed superiorly with pelvis directed medially. In this situation, pelvis of both kidneys is placed correctly as two kidneys fuse after completion of rotation on the vertical axis.
iii. Type C – Lump kidney. Here, unilaterally, fusion occurs over a wide surface and pelvises of both kidneys are placed anteriorly. The ureter from the ectopically positioned kidney crosses the midline and it is placed more inferiorly than the ipsilateral one.
iv. Type D – Tandem or L-shaped kidney. Here the crossed ectopic kidney lies transversely and fuses partly with the inferior part of the ipsilateral uncrossed kidney.
v. Type E – Disc kidney (unilateral in position). Here both the kidneys are fused along the whole medial surface.
vi. Type F – Superiorly fused and crossed kidney (least common type). Here the lower part of the crossed ectopically placed kidney fuses with the superior part of the ipsilateral uncrossed kidney. Pelvis of both the kidneys is anteriorly placed.

The ascent of primitive renal tissue (metanephric blastema) and ureteric bud starts in the fifth week of intrauterine life (IUL) which completes at ninth week of gestational age. Congenital anomalies of renal fusion and ectopia occur if there is impaired lateral shift, deviation, and internal rotation during the process of cephalic migration from the mid-pelvis to the abdomen due to:

i. Faulty ureteral bud development.
ii. Under influences of teratogenic factors.
iii. Aberrant renovascular phenotypes where an abnormally positioned umbilical artery causes opposition of the metanephric blastema resulting in a fusion anomaly. The retroperitoneal structures may impair the ascent of fused renal mass cranially up to the lumbar position.
iv. Cook and Stephens had proposed that abnormal flexion or growth of the developing hind-end may cause development of pancake kidney [9].

So, pancake kidney malformation is a result of extensive fusion of the medial surfaces of metanephric blastema during early IUL. The renal mass is commonly situated in the pelvis or at the level where aorta bifurcates [10]. A pancake kidney may get blood supply from branches of the abdominal aorta or from numerous branches of both the internal and external iliac arteries. Most commonly, inferior parts of the ectopic, fused kidneys are more medially rotated than superior parts. The pancake kidney is located commonly at the level of L3–L5 vertebra and lies in front of the great vessels. The parenchymatous or fibrous isthmus lies where the inferior mesenteric artery arises from the aorta [11].

Urogenital anomalies are found to be linked with 9p tetrasomy and 9p trisomy. Pancake kidney is often associated with other anomalies, such as undescended testis or anomalous vas deferens, vaginal agenesis, cornuate (uni/bi)uterus, Fallot’s tetralogy, spina bifida, agenesis of sacrum, caudal regression syndrome, and strabismus.

Pancake kidney mostly has a deformed and rotated collecting system. The shorter length of ureters cause obstruction and stasis leading to hydronephrosis, nephrolithiasis, and vesicoureteral reflux with recurrent infection of urinary tract. So, an otherwise asymptomatic pancake kidney, may present with vague lower abdominal pain, features of urinary tract infection like pyrexia and hematuria. Aneurysm of iliac vessel, amenorrhea, and failure to conceive may also be encountered as extrarenal manifestations. The pancake kidney is supplied by one or multiple renal arteries (branches from distal aortic or iliac artery) and is drained by renal veins (tributaries of iliac vein or inferior vena cava). In cases of single vascular supply, gravid uterus, pelvic mass, or trauma may lead to renal ischemia. Hypertension may result from stenosis of aberrant renal arteries due to atherosclerosis of the aorta and iliac arteries in the process of ageing [12].

In spite of the scarcity of reported cases of pancake kidneys, individuals with ectopic fused kidney anomalies are more inclined toward development of various primary malignancies, including renal cell carcinoma, Wilms tumor, or, rarely, rhabdomyosarcoma [13]. An individual with horseshoe kidney is nearly two times more prone to develop Wilms tumor in comparison to one with normal renal anatomy.

The diagnosis is always incidental. Excretory urography was used previously which is now replaced by USG, MDCT, computed tomography (CT) urography, and radio nucleotide scanning for better studies of the urinary system and renal vascular anatomy. USG is the primary modality for prenatal or postnatal diagnosis of kidney anomalies. CT urography with contrast enhancement (MDCT) is especially useful for studying urinary tract anatomy, which includes kidney parenchyma and collecting systems [14]. MDCT images can also be processed using multiplanar reconstruction methods, maximum intensity projection, and volume rendering process which provide three-dimensional imaging and help in better diagnosis. Crossed fused ectopic kidney has no specific treatment guidelines [15]. The mere existence of pancake kidney does not herald progressive renal failure. Surgery is only indicated in cases of confirmed failure or progressive derangement of kidney function due to urinary outflow obstruction/obstructive uropathy. The treatment is selective toward the associated problems like pyeloplasty for a pelvi-ureteric junction obstruction, bulking agent injection, or ureteric reimplantation in case of vesicoureteral reflux. To avoid vascular injury, infarction, necrosis, or postoperative renal failure, the fused renal mass should not be separated [10]. Conservative management is indicated for symptomless individuals, after exclusion of coexisting anomalies, in the form of regular monitoring of renal function, remaining alert for the development of recurrent infections, any obstructive (namely, ureteropelvic junction) manifestations leading to calculus formation, and any malignant changes. The individual should be careful in avoiding trauma to low-lying pelvic kidney. In the case of pregnancy in women with pancake kidney, possible compressive effect by gravid uterus and possible trauma during childbirth should also be kept in mind. During pelvic surgery, especially abdominal aortic surgery, the pancake kidney is also susceptible to iatrogenic trauma. As the blood supply to pancake kidney comes from aortic bifurcation or iliac vessels, the whole renal mass may suffer ischemia if proximal aortic cross-clamping is done [12].

To conclude, pancake kidney is the rarest anomaly occurring due to extensive fusion of medial surfaces of both the kidneys; it is commonly detected incidentally. There may be increased occurrence of recurrent urinary tract infections or renal calculus formation, due to the chances of ureteral obstruction by rotated and anomalous collecting systems. The pancake kidney can be managed conservatively, if the individual remains asymptomatic, by regular monitoring of renal function and remaining alert for the development of any malignant changes. The individual should be careful in avoiding trauma to low-lying pelvic kidney. Extensive surgeries are avoided and only selective procedures should be done so that the patient may lead a normal lifestyle.

## Data Availability

Presented within the manuscript, please contact author for additional data requests.

## Abbreviations

KUB: Kidney, ureter, and bladder
USG: Ultrasonography
MDCT: Multi-detector computed tomography
CT: Computed tomography
IUL: Intrauterine life
L3–L5: Third, fourth, and fifth lumbar vertebra
